# Increasing forest fire emissions despite the decline in global burned area

**DOI:** 10.1126/sciadv.abh2646

**Published:** 2021-09-24

**Authors:** Bo Zheng, Philippe Ciais, Frederic Chevallier, Emilio Chuvieco, Yang Chen, Hui Yang

**Affiliations:** 1 Institute of Environment and Ecology, Tsinghua Shenzhen International Graduate School, Tsinghua University, Shenzhen 518055, China.; 2 Laboratoire des Sciences du Climat et de l’Environnement, LSCE/IPSL, CEA-CNRS-UVSQ, Université Paris-Saclay, Gif-sur-Yvette, France.; 3 Climate and Atmosphere Research Center (CARE-C), The Cyprus Institute, 20 Konstantinou Kavafi Street, 2121 Nicosia, Cyprus.; 4 Environmental Remote Sensing Research Group, Department of Geology, Geography and the Environment, University of Alcalá, Calle Colegios 2, Alcalá de Henares 28801, Spain.; 5 Department of Earth System Science, University of California, Irvine, CA 92697, USA.; 6 Department of Biogeochemical Integration, Max Planck Institute for Biogeochemistry, 07745 Jena, Germany.

## Abstract

Satellites have detected a global decline in burned area of grassland, coincident with a small increase in burned forest area. These contrasting trends have been reported in earlier literature; however, less is known of their impacts on global fire emission trends due to the scarcity of direct observations. We use an atmospheric inversion system to show that global fire emissions have been stable or slightly decreasing despite the substantial decline in global burned area over the past two decades caused by the carbon dioxide emission increase from forest fires offsetting the decreasing emissions from grass and shrubland fires. Forest fires are larger carbon dioxide sources per unit area burned than grassland fires, with a slow or incomplete follow-up recovery—sometimes no recovery due to degradation and deforestation. With fires expanding over forest areas, the slow recovery of carbon dioxide uptake over burned forest lands weakens land sink capacity, implying positive feedback on climate change.

## Teaser

Global fire emissions have been rather stable over the past two decades despite a substantial decline in global burned area.

## INTRODUCTION

Global fires have widespread impacts on the global carbon cycle with immediate direct carbon emissions of about 2 gigatons (Gt) C year^−1^ (*1*) to the atmosphere and indirect legacy carbon sinks of previously burned lands. Fires affect land carbon sinks through ecosystem recovery of CO_2_ uptake after fires and pyrogenic carbon production, which can be a net sink of atmospheric carbon if this production increases, e.g., from more fires with a higher flaming temperature. About 20% of the global fire emissions represent a source (*2*) of CO_2_ that can be termed as irreversible on decadal to centennial time scales because it cannot be recovered by vegetation regrowth or soil carbon rebuild. This is the case for peatland fires, tropical deforestation, and degradation fires that have cascading effects on enhanced tree mortality and further carbon losses. Rainfall and temperature control the trends and interannual variability in fire regimes worldwide (*3*–*6*). Extreme drought events tend to induce abnormally large fire activities and fire emissions (*3*, *7*, *8*). In addition, fire activity is also altered by the interaction between ecosystem susceptibility and human influences on fires. For example, cropland and pasture expansion and fire suppression activities have continued to drive a significant decline in the global burned area from 1998 to 2015 (*9*).

Identifying the trends and drivers of fire emissions in each region is key to understanding the dynamic role of fires in shaping the terrestrial carbon balance. Unlike the global burned area that has been readily monitored by satellites (*10*), the amount of CO_2_ emitted from fires cannot be measured easily. The heterogeneity and variability in fire duration and intensity, combustion completeness and efficiency, and plant mortality with subsequent carbon decay all hamper the extrapolation of a few site measurements to a large spatiotemporal scale. Fire emission models based on remotely sensed fire activities provide global coverage with spatiotemporal detail but have large uncertainties in their emission estimates, as revealed by the large spread between models and their inconsistencies with observations (*11*, *12*). One alternative powerful approach is to quantify fire carbon emissions from satellite observations of atmospheric carbon monoxide (CO) (*7*, *13*), a key fire combustion tracer coemitted with CO_2_. Atmospheric inversion models combine transport modeling and CO observations to deduce time-varying maps of fire CO emissions. This approach has provided the opportunity of observing the fire CO emissions from the whole globe at a high temporal frequency for the past two decades. However, the spatial resolution and uncertainties in atmospheric inversions are limited by atmospheric transport models and satellite measurement. These long-term observations were made with the Measurements of Pollution in the Troposphere instrument (MOPITT) (*14*), a satellite providing remotely sensed CO concentrations globally. Previous analyses of fire CO_2_ emissions based on MOPITT only focused on specific years and regions and used static CO_2_-to-CO combustion ratios to convert CO emissions maps into CO_2_ emissions maps, without accounting for the spatial and seasonal variability in fire combustion efficiencies (*15*).

Here, we provide the first global reconstruction of fire CO and CO_2_ emissions from 2000 to 2019 based on the MOPITT CO observations. We combine these fire emission maps with the high-resolution burned area and land cover maps to evaluate the trends and drivers of fire emissions in the context of the global decline in burned area (*9*). First, we infer CO weekly emissions at a horizontal resolution of 3.75° × 1.9° from MOPITT version 8 CO column retrievals using an atmospheric Bayesian inversion system (*16*). Second, we establish empirical relationships between CO emission, combustion efficiency, and CO_2_ emission based on 127 field measurements from the literature (*17*). This allows us to convert MOPITT-based fire emissions of CO to CO_2_ considering variable combustion conditions on each inversion grid at a resolution of 3.75° × 1.9°. The conversion process uses the burned areas derived from the Global Fire Emissions Database (GFED 4.1s) and the Carnegie-Ames-Stanford Approach (CASA) model–simulated fuel loads to estimate fire CO emission factors and the associated combustion efficiencies (see Materials and Methods). Third, we attribute fire CO_2_ emission variations to the contributions of changes in burned areas and in emissions per unit of area burned using the 500-m resolution burned area data, which are remotely sensed by NASA’s satellite of Moderate Resolution Imaging Spectroradiometer (MODIS) (*10*). We also examine the spatial relationships between fire CO_2_ emissions and plant functional types (PFTs) derived from 300-m resolution land cover maps of the European Space Agency (ESA) (*18*) to investigate the influence of different biomes on fire emission dynamics. Last, we compare our emission estimates with fire model simulations from the Fire Model Intercomparison Project (FireMIP) (*19*) to understand model capabilities and limitations of those models that participated in FireMIP.

## RESULTS

### Trends and drivers of global fire emissions from 2000 to 2019

**Fig. 1.**
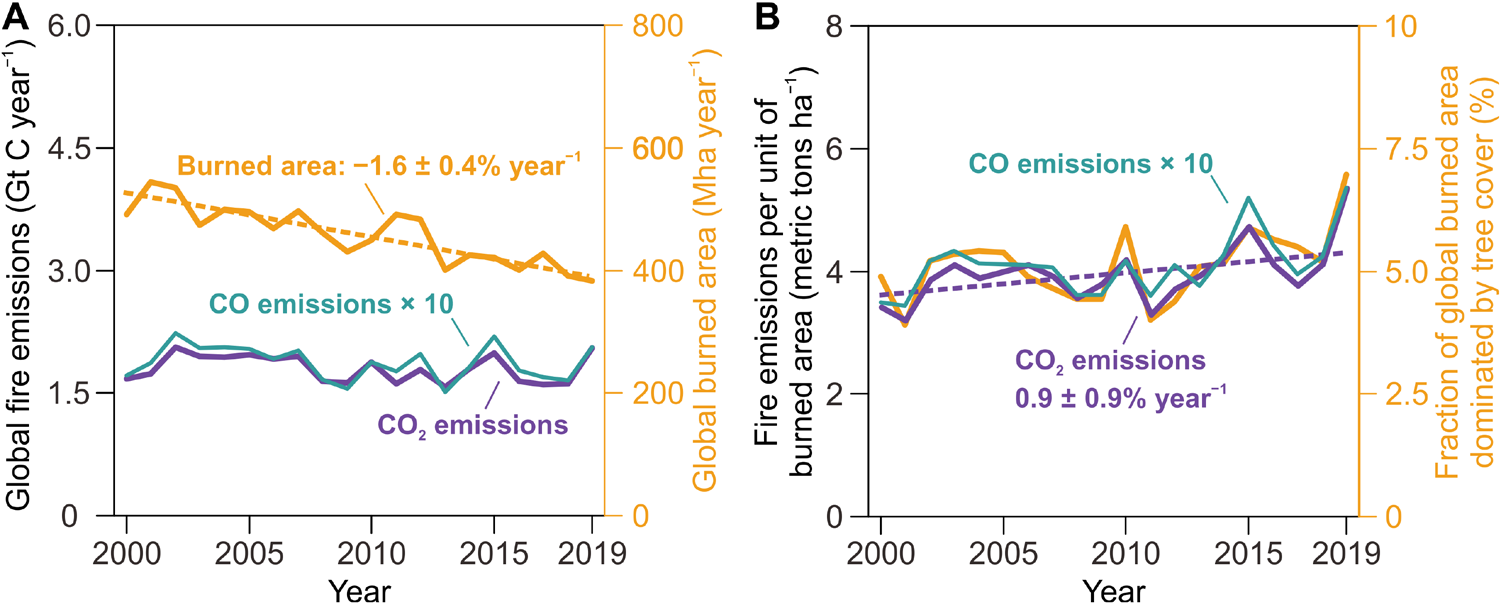
Global burned areas and fire emissions from 2000 to 2019. (**A**) Annual burned areas (orange curve) derived from MODIS 500-m resolution data product and the inversion-based estimates of fire CO (cyan curve) and CO_2_ (purple curve) emissions in this study. (**B**) Fraction of burned areas from the inversion model grid cells dominated by tree cover (orange curve) and the global average of the fire emissions of CO (cyan curve) and CO_2_ (purple curve) per unit of area burned. The values of trends are evaluated using the Mann-Kendall test.

On the basis of our analysis of the MOPITT CO observations and atmospheric inversions, we estimate the global fire CO_2_ emissions to be 1.8 Gt C year^−1^, on average, during 2000–2019, with a nonsignificant decreasing trend of −0.5 ± 0.8% year^−1^ (95% confidence interval; purple curve in Fig. 1A). The quasi-stable emissions combined with a significant decline in global burned areas (−1.6 ± 0.4% year^−1^; orange curve in Fig. 1A) suggest that the global mean emission intensity (i.e., CO_2_ emissions per unit of area burned) has increased by 0.9 ± 0.9% year^−1^ since 2000 (purple curve in Fig. 1B). Such an increasing emission intensity thus partially compensated for the decreasing trend in global burned areas and prevented a synchronous, rapid decline in the fire CO_2_ emissions. Global fire CO emissions, directly inferred from the CO atmospheric inversion, showed a slightly more negative but still insignificant decline of −0.7 ± 1.0% year^−1^ (cyan curve in Fig. 1A) than CO_2_ emissions, the emission intensity of CO increasing by 0.6 ± 0.9% year^−1^ since 2000 (cyan curve in Fig. 1B). With the use of our dynamic model to convert CO to CO_2_ emissions based on combustion conditions, the interannual variations of emission magnitudes and intensities are still consistent between CO_2_ and CO over the past two decades, probably implying the same underlying drivers. Figure S1 presents the global maps of prior and posterior fire fluxes of CO and CO_2_ derived from our atmospheric inversion and dynamic model results.

The contrast between satellite-based declining burned areas and inversion-based stable CO and CO_2_ emissions implies a global positive trend in the global mean fire emission intensity, which is crucial to understanding the drivers of global fire emission variations. On the basis of a geospatial analysis with ESA’s annual land cover maps (e.g., tree cover fraction shown in fig. S2), we find that interannual anomalies of fire emission intensities coincide regionally with a higher fraction of burned area from forest-dominated grid cells (i.e., trees cover more than 50% of the vegetated area within a model grid cell, which is different from the definition of forest in the Food and Agriculture Organization based on a tree canopy cover of more than 10% with trees capable of reaching a height of 5 m; orange curve in Fig. 1B). The coefficient of determination (*R*^2^) between these two factors between 2000 and 2019 is 0.77, suggesting that the changes in forest-dominated burned areas correlate highly with (probably explain 77% based on the *R*^2^ value) the interannual variability of global mean fire emission intensities. Although grid cells dominated by forest only account for 5% of global burned areas, they disproportionately contribute 20 and 18% of global CO and CO_2_ fire emissions, respectively. This is because trees have a larger fuel load and lose more carbon to the atmosphere than grasses per unit of area burned.

### Variation of fire activities and emissions across regions and biomes

**Fig. 2.**
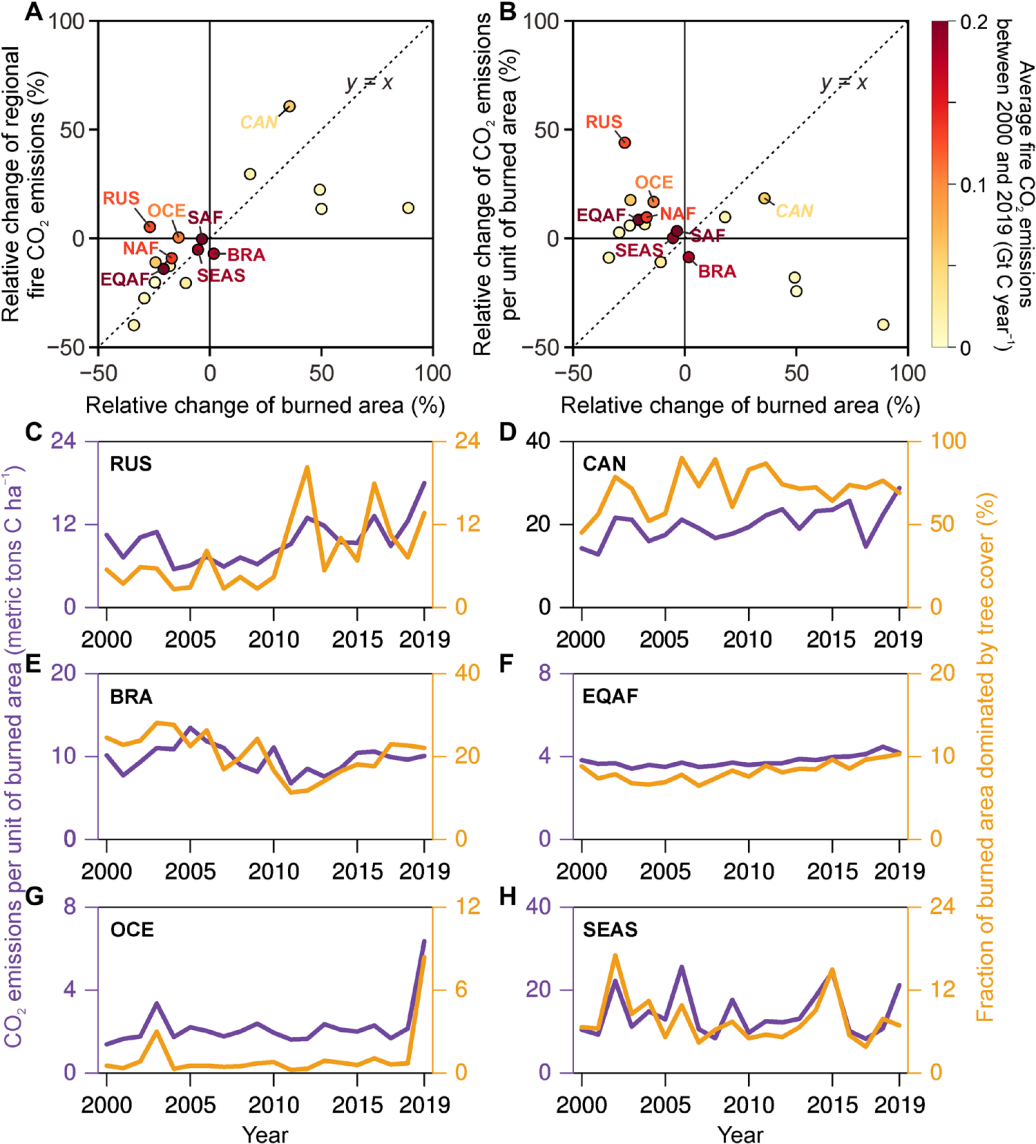
Regional trends in burned areas, fire CO_2_ emissions, and fire CO_2_ emissions per unit of area burned. Each dot in (**A**) and (**B**) represents a region according to fig. S3, with the color of the dots representing annual average fire CO_2_ emissions between 2000 and 2019. These dots are plotted according to the relative change in decadal average burned areas between 2000 to 2009 and 2010 to 2019 on the horizontal axis. The vertical axes represent the relative changes from 2000s to 2010s in fire CO_2_ emissions and fire CO_2_ emissions per unit of area burned in (A) and (B), respectively. (**C** to **H**) Regional trends in fire CO_2_ emissions per unit of area burned (purple curve) and the fraction of burned area from the inversion model grid cells dominated by tree cover (orange curve) from 2000 to 2019.

We next divide the globe into 18 fire regions (fig. S3) (*16*) to further understand the regional trends in burned areas, fire CO_2_ emissions, and emission intensities per unit of area burned (Fig. 2). Decadal changes (between 2000 to 2009 and 2010 to 2019) in fire CO_2_ emissions (Fig. 2A) were smaller than those of burned areas in regions such as Northern Africa (NAF) and Equatorial Africa (EQAF). In some regions where burned areas declined [e.g., Russia (RUS) and Oceania (OCE)], CO_2_ emissions showed an opposite increasing trend as a result of a concurrent growth of emission intensities (Fig. 2B). Canada and Alaska (CAN) is the region where both burned areas and emission intensities increased rapidly, driving a substantial increase in its fire CO_2_ emissions from the 2000s to the 2010s. Brazil (BRA) is the only region with little changes in burned areas but reduced emissions from the 2000s to 2010s, thus providing evidence for a reduction in fire emission intensities.

In all studied regions (Fig. 2, C to H), both the trends and the interannual variations of regional fire emission intensities are consistent with the changing fraction of burned area dominated by tree cover. The expansion of boreal forest fires in RUS (Fig. 2C) and CAN (Fig. 2D) increased fire emission intensities over these two regions. In BRA (Fig. 2E), the fire emission intensity increased from 2000 to 2005, decreased from 2005 to 2013, and later increased again through 2019. This pattern of variability corresponds to the slowing of Amazonian deforestation through the application of strict policies between 2005 and 2013 (*20*), followed by accelerated deforestation since then due to the rollback of those policies, which is broadly coincident with reported deforestation rates in BRA’s Amazon (*21*). In addition to these political interventions, the increase in fire incidence also shapes the fire emissions from Amazonian forests during drought years (*22*, *23*). In EQAF (Fig. 2F), the decline in burned areas led to small changes in the forest fire fractions and the emission intensities, because, in that region, fires occur predominantly in the savanna biome. In OCE (Fig. 2G), we observed an anomalously high fire emission intensity in 2019 due to the widespread bushfire in eastern Australia (*24*, *25*), a region that rarely burns. In Southeast Asia (SEAS) (Fig. 2H), the fire emission intensities are sensitive to drought events (*7*, *8*, *13*) with substantial interannual variations. The forest fires in RUS, Amazonia, Indonesia, and Australia presented simultaneous positive anomalies of fire emission intensity in 2019 (*26*, *27*). Consequently, the global fire CO_2_ emissions were 15% higher in 2019 than the 2000–2018 average, although the global burned areas were observed at the lowest level in 2019, in line with the long-term decreasing trend (Fig. 1A). Such a compound event with high forest fires in several regions is a source of concern for the stability of the land carbon sink.

**Fig. 3.**
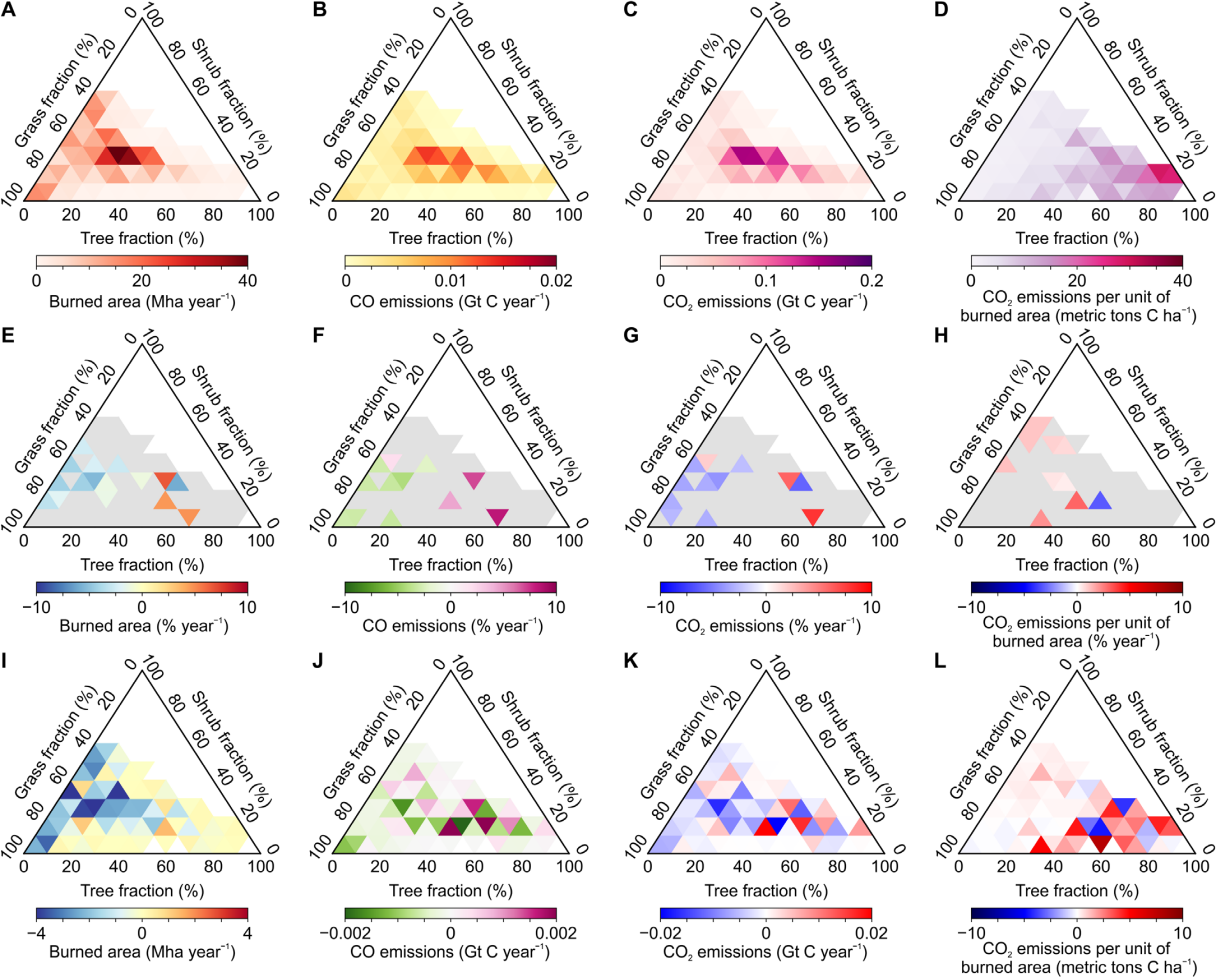
Spatiotemporal dynamics of burned areas, fire CO and CO_2_ emissions, and CO_2_ emissions per unit of area burned. Ternary plots are used to depict the spatial distribution patterns across tree, shrub, and grass cover fractions in the vegetated area within an inversion model grid cell. The plots of the first row (**A** to **D**) show the annual averages between 2000 and 2019, the plots of the second row (**E** to **H**) show the trends from 2000 to 2019 estimated by the Mann-Kendall test with the gray color representing a lack of statistically significant trends, and the plots of the third row (**I** to **L**) show the change in decadal averages between 2000 to 2009 and 2010 to 2019. The plots of the first column (A, E, and I) are for burned areas, the plots of the second column (B, F, and J) are for fire CO emissions, the plots of the third column (C, G, and K) are for fire CO_2_ emissions, and the plots of the fourth column (D, H, and L) are for fire CO_2_ emissions per unit of area burned.

We also analyzed ESA’s annual land cover maps (*18*) with MODIS-observed burned areas (*10*) and our MOPITT-constrained fire emission estimates using a grid-based approach. The land cover of each inversion model grid (3.75° longitude × 1.9° latitude) is categorized into tree, shrub, and grass fractions in the vegetated areas, and the spatial connections between burned areas and fire emissions are illustrated on the basis of those three land cover types (Fig. 3). The grassland vegetation dominates both the global burned areas (Fig. 3A) and the declining trends in burned areas since 2000 (Fig. 3, E and I). In contrast, the burned areas present statistically significant increasing trends in the grid cells, with a tree cover fraction larger than 50%. The biomes that exhibit statistically significant trends in burned areas did not show statistically significant trends in CO_2_ emission intensities (Fig. 3H). Consequently, the grass-dominated lands significantly reduced fire emissions of both CO (Fig. 3F) and CO_2_ (Fig. 3G), while the tree-dominated lands tended to increase fire emissions. On a decadal scale from the 2000s to 2010s, although large interannual variations existed, we observe more fire emissions from the tree cover–dominated grid cells but fewer emissions from grassland grid cells (Fig. 3, J and K). To confirm this, we also integrate our inversion-based gridded fire emissions with the global forest change product of Hansen *et al*. (*28*) and the driver map of global forest loss from Curtis *et al*. (*29*) (fig. S4), suggesting that the increase in fire CO_2_ emissions mainly occurred over grid cells affected by tree loss. The analysis further suggests that the increase in forest fire emissions was not primarily caused by commodity-driven deforestation and shifting agriculture over the past decade. The tendency toward more forest burning drove up the global average fire emission intensities, which led to the distinct fire emission trends between grasslands and forests and lastly caused the decoupling of burned area trends and fire emission trends at the global scale.

### Comparison with FireMIP model simulations

**Fig. 4.**
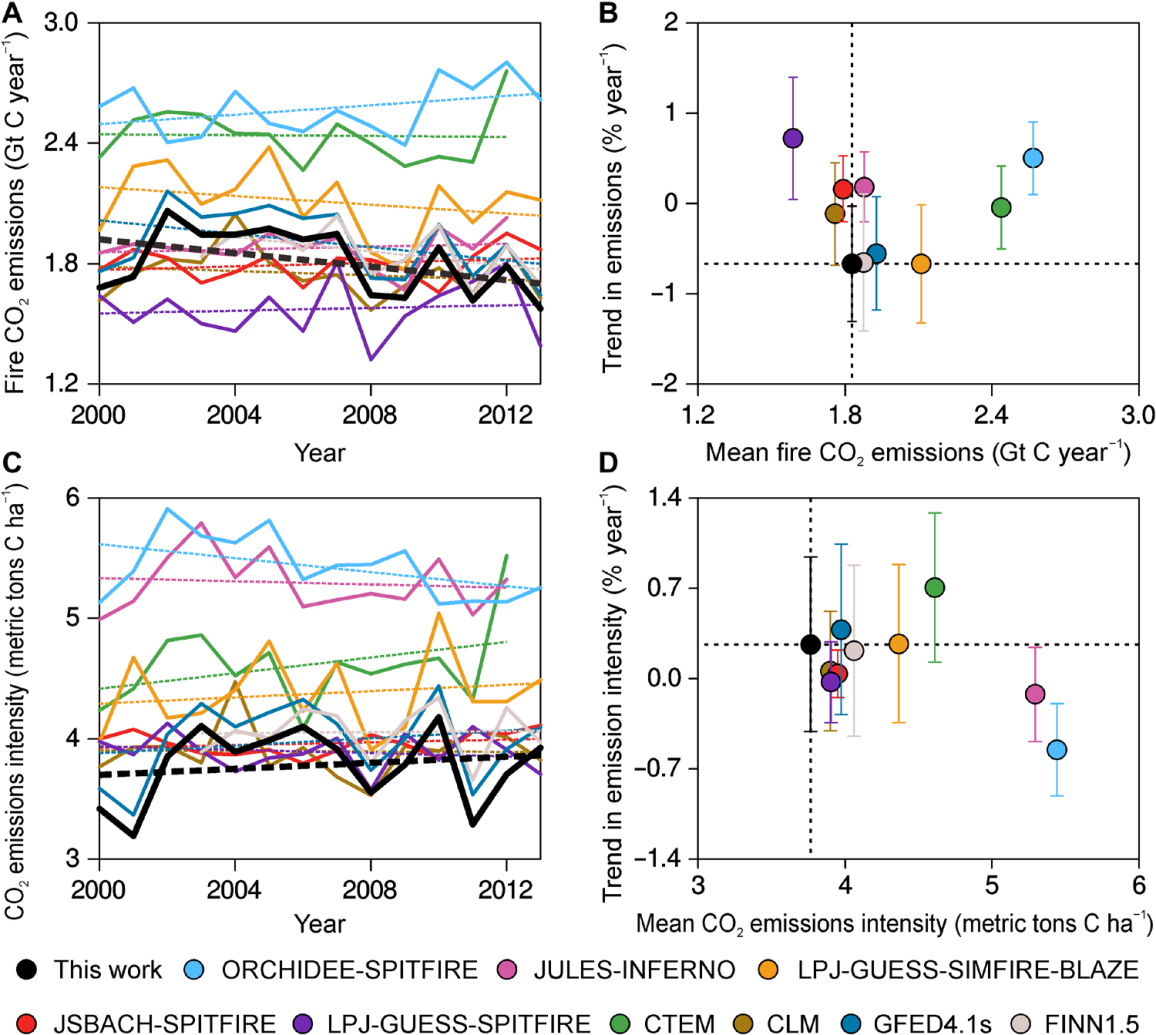
Comparisons of the FireMIP model simulations, GFED 4.1s, and FINN1.5 emissions with the inversion-based estimates. (**A** and **B**) Global fire CO_2_ emissions from 2000 to 2013 (several FireMIP model simulations are available up to 2012) and the trends from 2000 to 2012, respectively. (**C** and **D**) Global average fire CO_2_ emissions per unit of area burned from 2000 to 2013 and the trends from 2000 to 2012, respectively. The error bars in (B) and (D) are the range of ±1 SD that represents uncertainties in the trend estimation. The black curves and dots represent the inversion-based emission estimates in this study, and the other curves and dots are derived from the FireMIP models (*19*, *38*), GFED 4.1s (*1*), and FINN1.5 (*39*). Here, we only use the simulation results from seven of the nine models in FireMIP. Two models were discarded, because they estimated more than 80% lower global burned areas than the others.

The global fire modules incorporated into terrestrial biosphere models from the FireMIP project (*30*) do not capture the decadal trends in fire CO_2_ emissions nor the fire emission intensity trends (Fig. 4). The seven FireMIP models shown in Fig. 4 gave global fire CO_2_ emissions of 1.6 to 2.6 Gt C year^−1^, on average, from 2000 to 2012 and simulated CO_2_ emission intensities within the range of 3.9 to 5.4 metric tons C ha^−1^. Our inversion-based analysis estimated 1.8 Gt C year^−1^ emissions and 3.8 metric tons C ha^−1^ for emission intensities during the same period. Besides, the inversion-based emission estimates present small declines in CO_2_ emissions at a rate of −0.7 ± 0.6% year^−1^ (black dot in Fig. 4B) from 2000 to 2012, the trend of which is missed by the FireMIP models. The MOPITT-based inversions combined with MODIS burned areas produce an increasing trend of 0.3 ± 0.7% year^−1^ from 2000 to 2012 in global mean fire emission intensity (black dot in Fig. 4D); however, only two FireMIP models simulated such an increasing trend. Three models even simulated decreasing trends in fire emission intensities, probably because they did not reproduce the tendency of the decreasing fires in grassland regions and the increasing fires in forest regions. Therefore, the representation of spatiotemporal dynamics of fires in different biomes needs to be improved in the fire models. The previous literature also shows that the FireMIP models cannot capture the observed decline in global burned area (*9*).

## DISCUSSION

The major uncertainties in our study could lie in (i) the mismatch in spatial resolutions between atmospheric inversions and explanatory variables (e.g., burned area and land cover) and (ii) the uncertainties underlying these data. First, our conclusions on emission trends and drivers are not affected by the discrepancy in spatial resolutions of data. To characterize emission drivers across biomes, we estimate the fraction of vegetation cover in each inversion model grid cell and investigate the spatial variation of fire emissions with vegetation cover composition (Fig. 3). Such an analysis leads to the same conclusion as the large-scale regional analysis (Fig. 2), both suggesting that the contribution of fire emissions from tree-dominated lands has been increasing in recent years. This phenomenon has also been confirmed by an independent dataset of global forest change (fig. S4). Second, the uncertainties in atmospheric inversions have been evaluated in our previous study (*16*), which shows that the trends of inversion emissions were robust to different observation constraints, prior fluxes, and hydroxyl radical fields. The magnitudes of global burned areas differ among different data products while they present consistent declining trends since 2000 (fig. S5). Another source of uncertainty comes from the approximation of small fires from 2017 to 2019 based on the ratio map of GFED 4.1s to MODIS burned areas averaged between 2000 and 2016 (fig. S6). The assumption of no time dependency in the ratio map could cause uncertainties at grid scale, while the small interannual variability in the ratios constrains the uncertainties in the analysis of burned areas at global and regional scales, which is not expected to influence the trend analysis in this study.

This study suggests that although global burned areas have declined over the past two decades, the carbon emissions from global fires were not reduced proportionally. The declining CO_2_ emissions from the reduced burned areas over grasslands, mainly in African savannas, were partially compensated by larger fire emissions caused by the tendency of increased forest burning, particularly in the boreal and Amazonia ecosystems. Those opposite trends have been retrieved from the satellite observations of CO concentrations over different biomes and can be interpreted through the atmospheric inverse modeling in terms of the spatiotemporal dynamics of fire CO_2_ emissions. However, the current generation of fire models does not capture these trends, which reveals substantial gaps in our understanding of fire dynamics and drivers for different biomes. The increasing area burned over forests and the inability of models to reproduce this behavior over the study period implies that realistically projecting future fire emissions is difficult. Unlike grassland fires, forest fires account for a large part of the net irreversible source of fire CO_2_ emissions. More forest fires without rapid recovery weaken the land carbon sink capacity in the following years, indicating that pressures from fires on climate have not been relieved despite the decline in global burned area. We need to extend our ability not only to monitor an anomaly of fire emissions but also to track the decreased land carbon sink after fires, which could be monitored by satellites observing the recovery over forests.

## MATERIALS AND METHODS

### Global fire CO emissions inferred from atmospheric inversions

We use a global atmospheric inversion system developed within our previous study (*16*) with improved data input to estimate the global fire CO emissions from 2000 to 2019. This inversion system is built upon the global three-dimensional transport model of the Laboratoire de Météorologie Dynamique (LMDz) coupled with the Simplified Atmospheric Chemistry Assimilation System (SACS) (*31*, *32*) and can infer the surface fluxes of trace gases from observations based on the Bayes’ theorem (*33*). This model framework has been developed and maintained by the research group at the Laboratoire des Sciences du Climat et de l’Environnement for more than 10 years, with the latest version developed and applied to reconstruct the global atmospheric CO budget from 2000 to 2017 (*16*) constrained by the MOPITT version 7 retrievals of CO columns. The optimized CO budget corrected the modeling bias of the prior data and agreed well with the independent measurement of surface CO concentrations. The trends of the optimized CO emissions were robust to different observation constraints, prior interannual variation, and the trends of hydroxyl radical according to the sensitivity analysis and matched the independent estimates from accurate regional inventories (*16*). In this study, the LMDz-SACS inversion system is further improved in two aspects. First, the meteorological field is nudged to the ERA5 global reanalysis (*34*), a new generation European Centre for Medium Range Weather Forecasts atmospheric reanalysis product that replaces the ERA-Interim data. Second, we use the MOPITT version 8 data as an observational constraint, which reduces the long-term bias drift and geographically variable retrieval bias compared to the previously used version 7 data (*14*).

With the improved inversion system, we estimated the global fire CO emissions from 2000 to 2019 based on the method of our previous studies (*7*, *15*, *16*). We first inferred surface total fluxes of CO from the MOPITT satellite CO retrievals at a spatial resolution of 3.75° longitude × 1.9° latitude every 8 days. Then, the CO total fluxes were split into three sources on land (i.e., anthropogenic, biomass burning, and biogenic) and the oceanic source based on the distinct spatial seasonal distributions of CO emissions from these four different sources. Anthropogenic sources are incomplete combustion processes of fossil fuels and biofuels. Biomass burning sources are the fires caused by humans or lightning on fire-prone landscapes such as grasslands and forests. Biogenic sources refer to the CO emissions generated by plant leaves. The oceanic sources release CO to the atmosphere from marine biogeochemical cycling. The proportions of emissions from each source in the model grids (3.75° × 1.9°) are derived from the latest prior emission inventories (*16*). Since fires dominate the local CO emissions in the fire season and the trend analysis cancels out a major part of the systematic errors, the reconstruction of the fire emissions from our atmospheric inversions constrains the attribution bias of source-specific CO fluxes. In this work, we analyzed the fire emissions at an aggregated level (by region and land cover type), which further reduces the uncertainties in fire emission estimates at grid scale.

### Global fire CO_2_ emissions derived from dynamic CO_2_-to-CO ratios

Our estimate of fire CO_2_ emissions is based on the inversion-based fire CO emissions and on the modeling of the modified combustion efficiency (MCE). MCE is defined as the ratio of carbon in fire CO_2_ emissions to carbon in fire emissions from both CO and CO_2_. MCE is a key metric that represents burning efficiency in vegetation fires, reflecting the relative role of flaming and smoldering combustions (*17*). Following the definition of MCE, we write the CO_2_ fire emissions as

$$E_{\text{CO}2,i,j}=\frac{{\text{MCE}}_{i,j}}{1-{\text{MCE}}_{i,j}}\times E_{\text{CO},i,j}\times \frac{44}{28}$$

where *i* represents a month between 2000 and 2019, *j* represents a model grid cell, *E*_CO2_ and *E*_CO_ are the fire emissions of CO_2_ and CO, respectively. The spatiotemporal dynamics of MCE are reconstructed using the method established by our previous study (*15*), where MCE was written as a linear function of CO emission factors based on the field measurements and the CO emission factors were derived from inversion emissions combined with a fire fuel combustion database.

Specifically, MCE and fire CO emission factors are estimated by the following two equations

$${\text{MCE}}_{i,j}=a+b\times {\text{EF}}_{\text{CO},i,j}$$

$${\mathrm{EF}}_{\text{CO},i,j}=\frac{E_{\text{CO},i,j}}{{\text{BA}}_{i,j}\times {\text{Fuel}}_{i,j}}$$

where EF_CO_ is the fire CO emission factor, *a* and *b* are the coefficients that determine the relation between MCE and EF_CO_, BA is the burned area, and Fuel is the biomass combustion per unit area burned. The coefficients *a* and *b* are estimated using the robust linear regression method based on 127 sets of field measurements collected by Andreae (*17*), including the measurement of fires in savanna and grassland (sample number: 31), boreal forest (13), temperate forest (39), tropical forest (9), peatland (6), and agricultural residues in fields (29). All of these measurement data and the regression line are presented in fig. S7. The *R*^2^ of the linear regression is 0.94, indicating the robustness of this model that predicts MCE based on EF_CO_. *a* and *b* are estimated as 0.9971 and −0.0009, respectively, which are consistent with other estimates from the literature (*15*, *35*). We use this linear model built upon wildfire measurement campaigns for different biome categories to estimate the gridded monthly MCE from 2000 to 2019 over the globe. The monthly maps of BA and Fuel are both derived from the fourth version of the GFED 4.1s (*1*), which retrieved BA accounting for small fires and estimated Fuel using the CASA model with some necessary adjustments based on field measurements and literature values. For the years from 2017 to 2019, the GFED 4.1s only provided a beta version of the estimate of biomass combustion (i.e., BA × Fuel) when this study was conducted but has not given the BA and Fuel values separately.

### Burned area and land cover maps

The burned areas used in this study are derived from the MODIS 500-m resolution burned area product (MCD64A1) (*10*) after implementing the corrections to account for small fires. The coarse resolution satellite data could lead to omission errors in the MODIS-observed burned areas (*36*, *37*). The GFED 4.1s dataset develops algorithms to estimate the burned areas of small fires, and the comparison between MODIS and GFED 4.1s burned area suggests that the MODIS data are slightly lower than the GFED 4.1s one, probably due to the omission of small fires in MODIS. To correct the underestimation bias, we build a map (fig. S6) representing the ratios of GFED 4.1s burned areas to MODIS ones at the spatial resolution of 3.75° × 1.9° using the multiannual average between 2000 and 2016 when the two datasets are both available. This map is used to adjust the MODIS burned area data from 2017 to 2019 to create consistent burned area maps as the GFED data used in this analysis. Besides, this study mainly focuses on the analysis of decadal trends, which are not expected to be affected by the difference in absolute values of burned areas between the MODIS and GFED 4.1s estimates.

The global land cover maps used in this study are derived from the 300-m resolution annual land cover product produced by the ESA Climate Change Initiative, which divides the land surface into 37 classes at a spatial resolution of 300 m from 1992 to 2018. We use the tool developed by Li *et al*. (*18*) to translate the 37 original land cover classes into 14 different PFTs, which are further aggregated into three vegetated categories (i.e., tree, shrub, and grass) and a nonvegetated category. The 2019 data are not available at present; therefore, the 2018 data were used instead. All of the PFTs maps are aggregated into the spatial resolution of 3.75° × 1.9° in our analysis. To illustrate the spatial correlation between fire emissions and tree loss, we also use the global forest change product (30 m × 30 m) from Hansen *et al*. (*28*) and the driver map of global forest loss (0.1° × 0.1°) from Curtis *et al*. (*29*), which are aggregated into the resolution of 3.75° × 1.9° in the analysis.
