# Supplementary material for: Increasing forest fire emissions despite the decline in global burned area
Source: Sci Adv. 2021 Sep 24;7(39):eabh2646. doi: 10.1126/sciadv.abh2646 (PMC8462883; doi:10.1126/sciadv.abh2646)
Supplement: Supplementary file 1 — Figs. S1 to S7 [file sciadv.abh2646_sm.pdf]

Science Advances
AAAS

Supplementary Materials for

Increasing forest fire emissions despite the decline in global burned area

Bo Zheng*, Philippe Ciais, Frederic Chevallier, Emilio Chuvieco, Yang Chen, Hui Yang

*Corresponding author. Email: bozheng@sz.tsinghua.edu.cn

Published 24 September 2021, *Sci. Adv.* **7**, eabh2646 (2021)
DOI: 10.1126/sciadv.abh2646

This PDF file includes:

Figs. S1 to S7

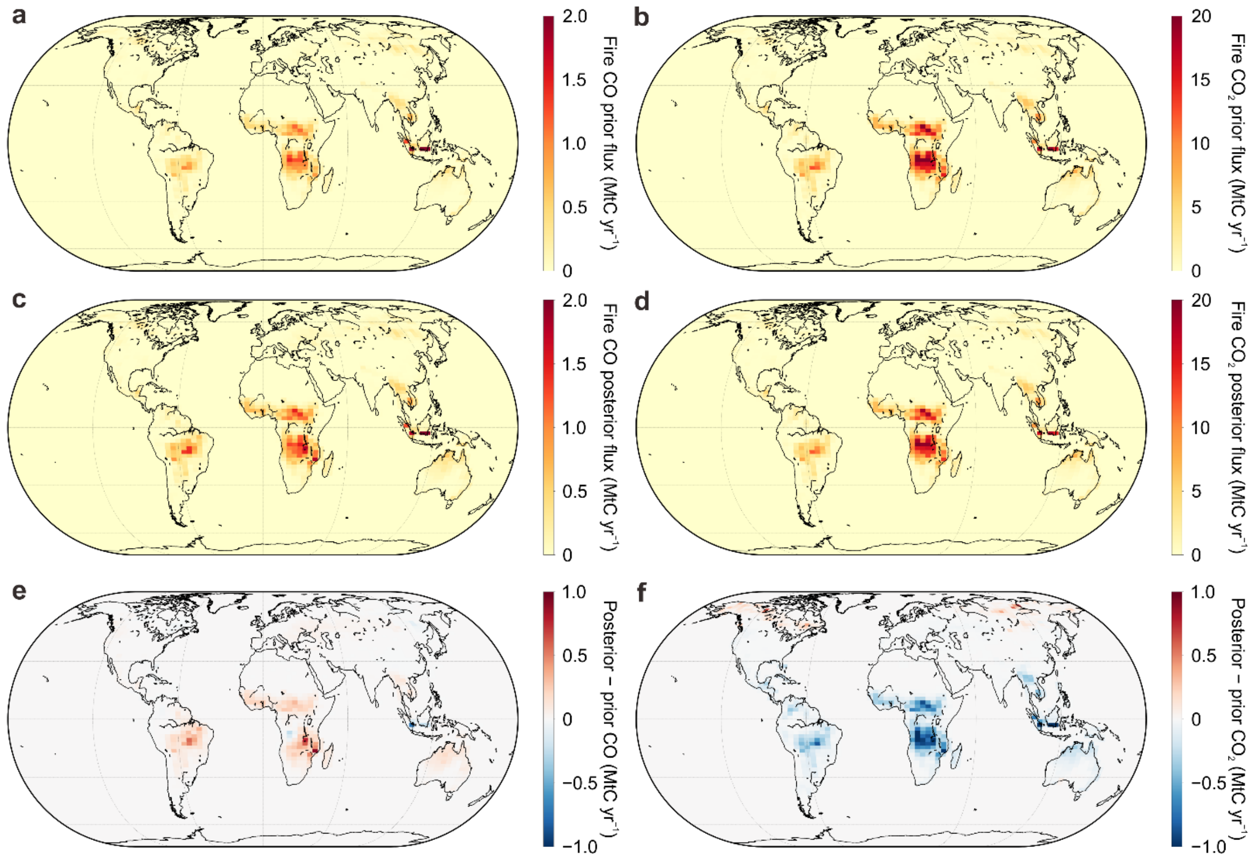

Fig. S1. Global fire CO and CO_2 emission fluxes averaged between 2000 and 2019. (**a**) and (**b**) show the prior fluxes of fire CO and CO_2, respectively. (**c**) and (**d**) show the posterior fluxes of fire CO and CO_2, respectively. (**e**) and (**f**) show the differences between posterior and prior fluxes for CO and CO_2, respectively. All of the data have a spatial resolution of 3.75° × 1.9°, derived from the atmospheric inversions in this study.

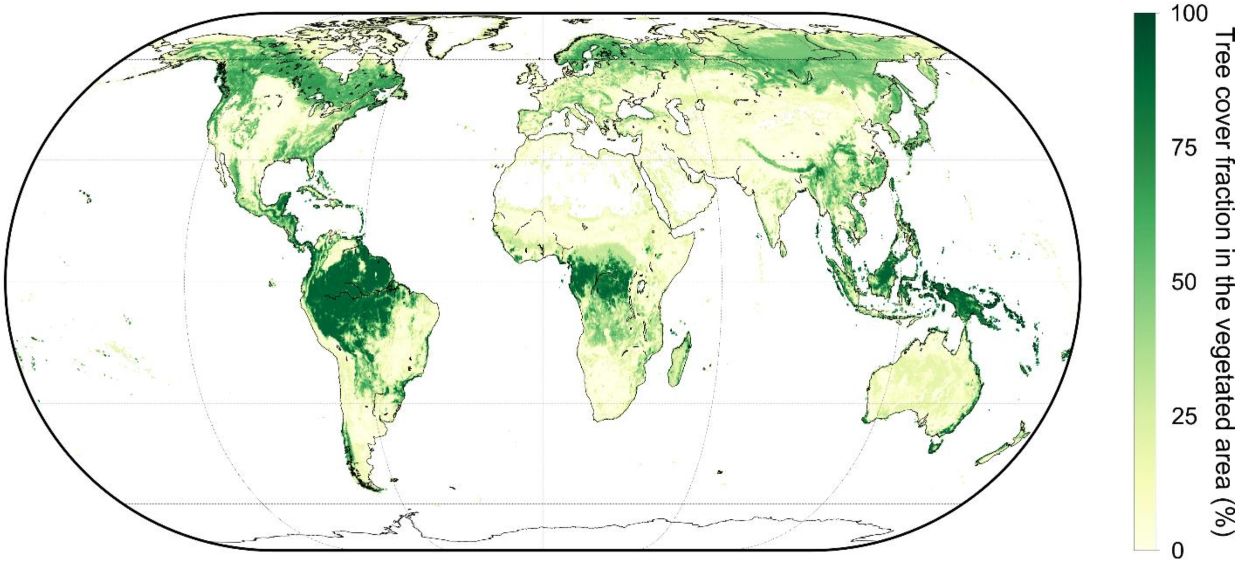

Fig. S2. Tree cover fraction in the vegetated area averaged between 2000 and 2018. The data have a spatial resolution of 0.25° × 0.25°, which are derived from ESA's 300-m resolution annual land cover maps.

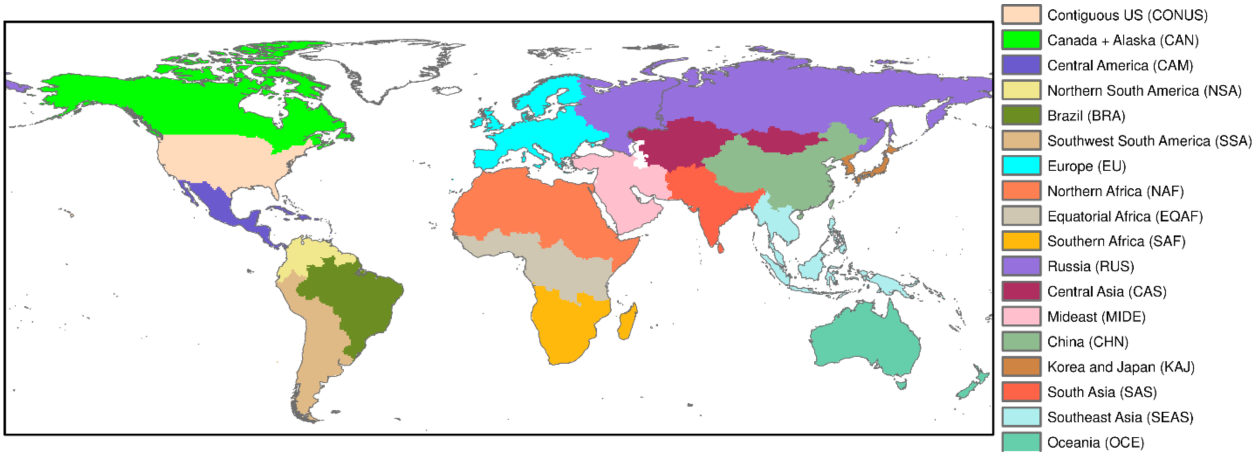

Fig. S3. The 18 regions used in the regional analysis.

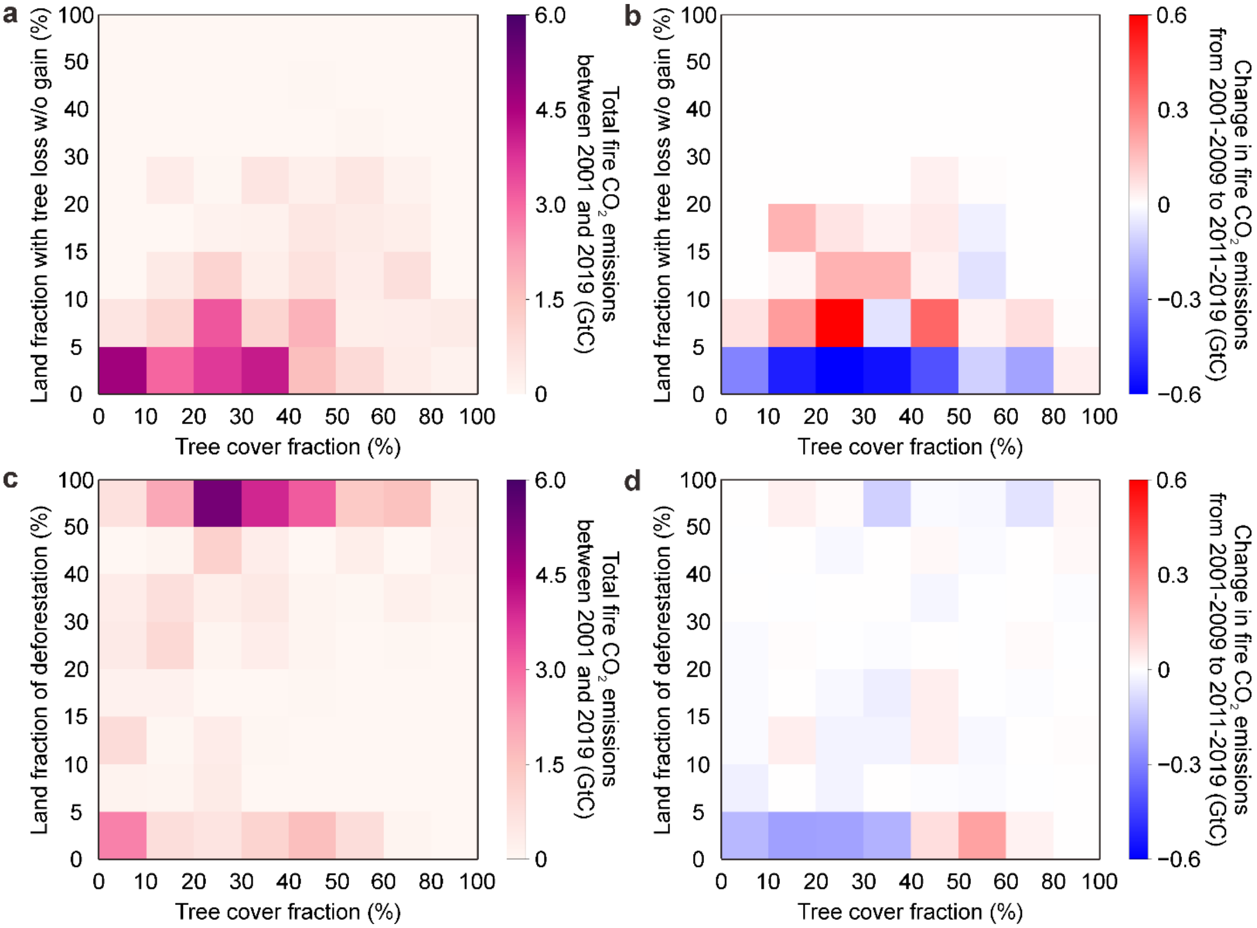

Fig. S4. Influence of tree loss and deforestation on global fire CO_2 emissions. (**a**) and (**c**) show the total fire CO_2 emissions between 2001 and 2019; (**b**) and (**d**) show the change in decadal total fire CO_2 emissions from 2001–2009 to 2011–2019. The x-axes represent the tree cover fraction derived from the ESA annual land cover maps. The y-axes of (**a**) and (**b**) represent the land fraction of tree loss without tree cover gain in inversion model grid cells based on the global forest change product of Hansen et al. (*28*). The y-axes of (**c**) and (**d**) represent the land fraction of commodity-driven deforestation and shifting agriculture in inversion model grid cells derived from Curtis et al. (*29*).

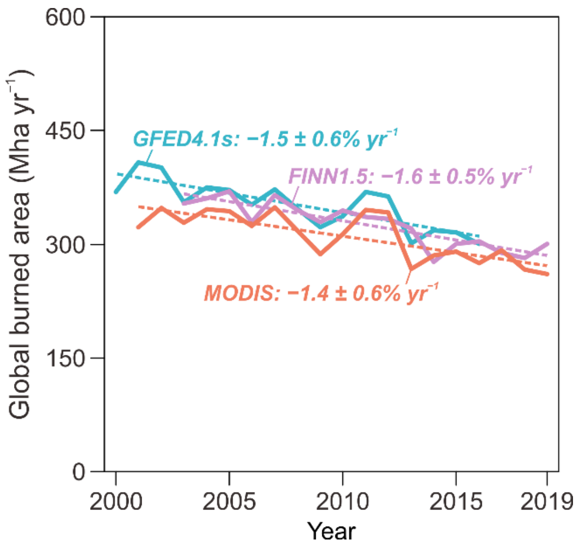

Fig. S5. Global burned areas since 2000 derived from GFED4.1s, FINN1.5, and MODIS.

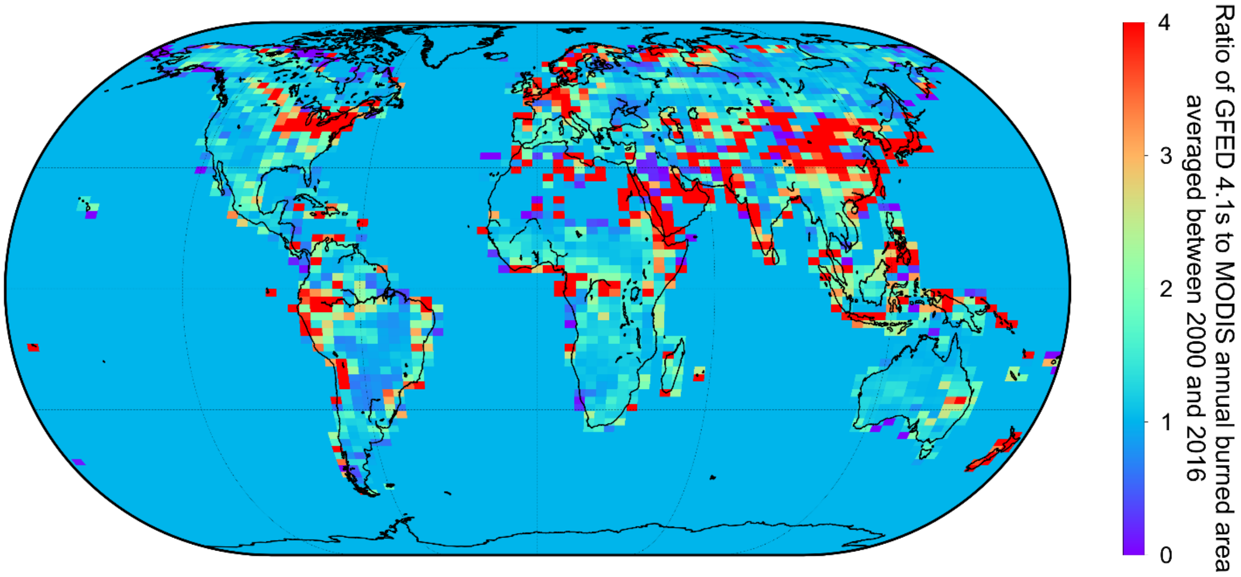

Fig. S6. The ratio of GFED 4.1s to MODIS annual burned area averaged between 2000 and 2016. The ratio map presented here has a spatial resolution of 3.75° × 1.9°.

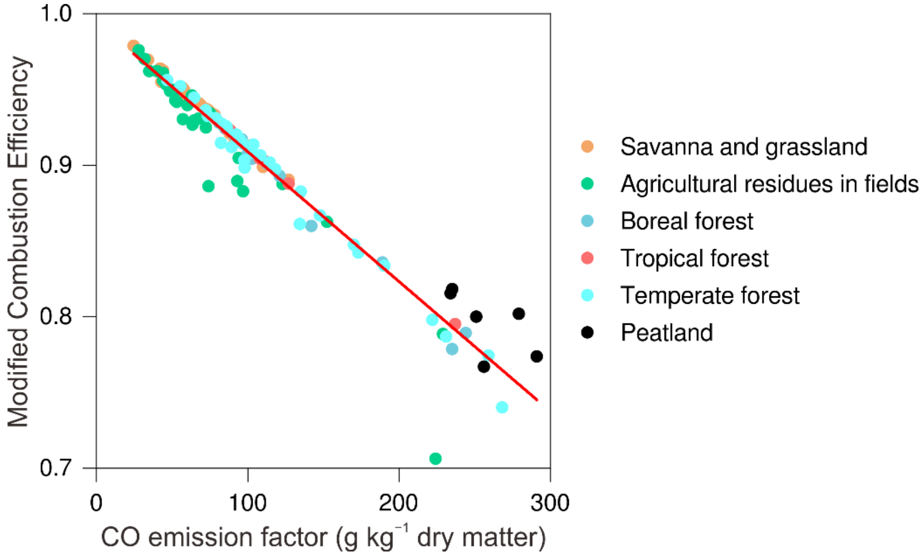

Fig. S7. The 127 sets of field measurement data used for relating fire CO emission factor with modified combustion efficiency.
